# Influence of oral comprehensive nursing intervention on mechanically ventilated patients in ICU: a randimized controlled study

**DOI:** 10.1186/s12912-023-01464-w

**Published:** 2023-08-29

**Authors:** Shengxia Lei, Yan Liu, Enkun Zhang, Chuanxia Liu, Jing Wang, Lingling Yang, Ping Zhang, Ying Shi, Xiaomin Sheng

**Affiliations:** Department of Critical Medicine, Funan County People’s Hospital, Fuyang, Anhui Province China

**Keywords:** Oral care, ICU, Mechanical ventilation

## Abstract

**Objective:**

To explore the effect of oral comprehensive nursing intervention on mechanically ventilated patients in ICU.

**Methods:**

Select 76 cases of mechanically ventilated patients in severe ICU admitted to our hospital from January 2022 to October 2022 as the research objects, and divide them into the control group and the observation group according to the way the patients receive oral care. 38 cases each. The patients in the control group received routine nursing intervention, and the patients in the observation group received comprehensive oral nursing intervention on the basis of the nursing of the control group. The clinical index data, oropharyngeal hygiene, pH value, blood gas analysis index levels, and the occurrence and death of ventilator-associated pneumonia were compared between the two groups of patients.

**Results:**

The hospitalization time of the two groups was compared (P > 0.05); the mechanical ventilation time and ICU stay time of the observation group were significantly lower than those of the control group (all, P < 0.05); the oral odor scores, The plaque index and soft scale index were significantly lower than those of the control group (all, P < 0.05); the pH value, PaO _2_ value, and SpO _2_ value of the observation group were significantly lower than those of the control group, and the PaCO _2_ value was significantly higher than that of the control group. group (all, P < 0.05); the incidence of VAP in the control group was 55.26%, and the mortality rate was 15.79%, the incidence rate of VAP in the observation group was 21.05%, and the mortality rate was 2.63%, and the incidence rate and mortality rate of VAP in the observation group were significantly lower in the control group (all, P < 0.05).

**Conclusion:**

The application of nursing intervention can effectively promote the recovery of patients, improve the hygiene of patients’ oropharynx, adjust the levels of pH and blood gas-related indicators in patients, and reduce VAP in patients. risk of morbidity and mortality.

## Introduction

In recent years, with the development of clinical intensive care medicine, mechanical ventilation (MV) orotracheal intubation has been widely used in intensive care unit (ICU) [1]. Critically ill patients usually have a long course of disease and are relatively critically ill. Most patients have low or even lost self-care ability, and their body’s resistance is also lower than that of normal people [2]. Studies [3, 4] have shown that operations such as endotracheal intubation, nasal feeding, and the use of antibiotics may lead to changes in the oral environment of patients and a decrease in saliva secretion. The combination of the above factors will make it difficult to clean the patient’s mouth thoroughly, resulting in the occurrence of residual impurities and dental plaque, which will lead to the occurrence of complications such as bad breath and oral ulcers, which will further aggravate the patient’s discomfort and pain. Ventilator-associated pneumonia (VAP) [4]. In recent years, clinical research on VAP [ 5 ] has confirmed that the oral hygiene status of patients is directly related to the occurrence of VAP. Studies have reported that 5–40% of patients requiring mechanical ventilation are affected by VAP, depending on the country, type of intensive care unit (ICU) and diagnostic criteria for VAP, which is associated with higher all-cause mortality and longer mechanical ventilation and ICU stay [1].

Previous studies [5, 6] found that scientific and efficient oral care interventions can effectively improve the oropharyngeal hygiene of ICU patients undergoing mechanical ventilation, thereby reducing the risk of VAP in patients. A study [7] showed that high-quality oral care intervention can reduce the incidence of VAP in patients by 33.3%. The above research shows that oral care, as a routine daily nursing measure for ICU patients undergoing mechanical ventilation, plays an important role in the various nursing programs for the prevention of VAP [8].

Discontinuation is recommended when current practices have been shown to be ineffective or harmful, or when the potential harms outweigh the benefits, defined as discontinuing the use of a medical practice after a previously adopted one. Studies have shown that routine practices that indicate discontinuation continue to exist despite evidence of limited benefit or potential harm. For example, the perception by clinicians that chlorhexidine provides significant benefits may lead to concerns about discontinuing this treatment and the need for alternative action. Suggested strategies to address this phenomenon include conducting rigorous cancellation trials to eliminate one intervention while advancing an alternative that is ethical and evidence-based in practice (i.e. a standardised oral care). Outcomes of concern should then be measured and reported to stakeholders.

Based on the above reasons, this study attempts to apply oral comprehensive nursing intervention to ICU mechanically ventilated patients, which aims to explore the impact of oral comprehensive nursing intervention on ICU mechanically ventilated patients.

## Objects and methods

### Research object

**Fig. 1:**
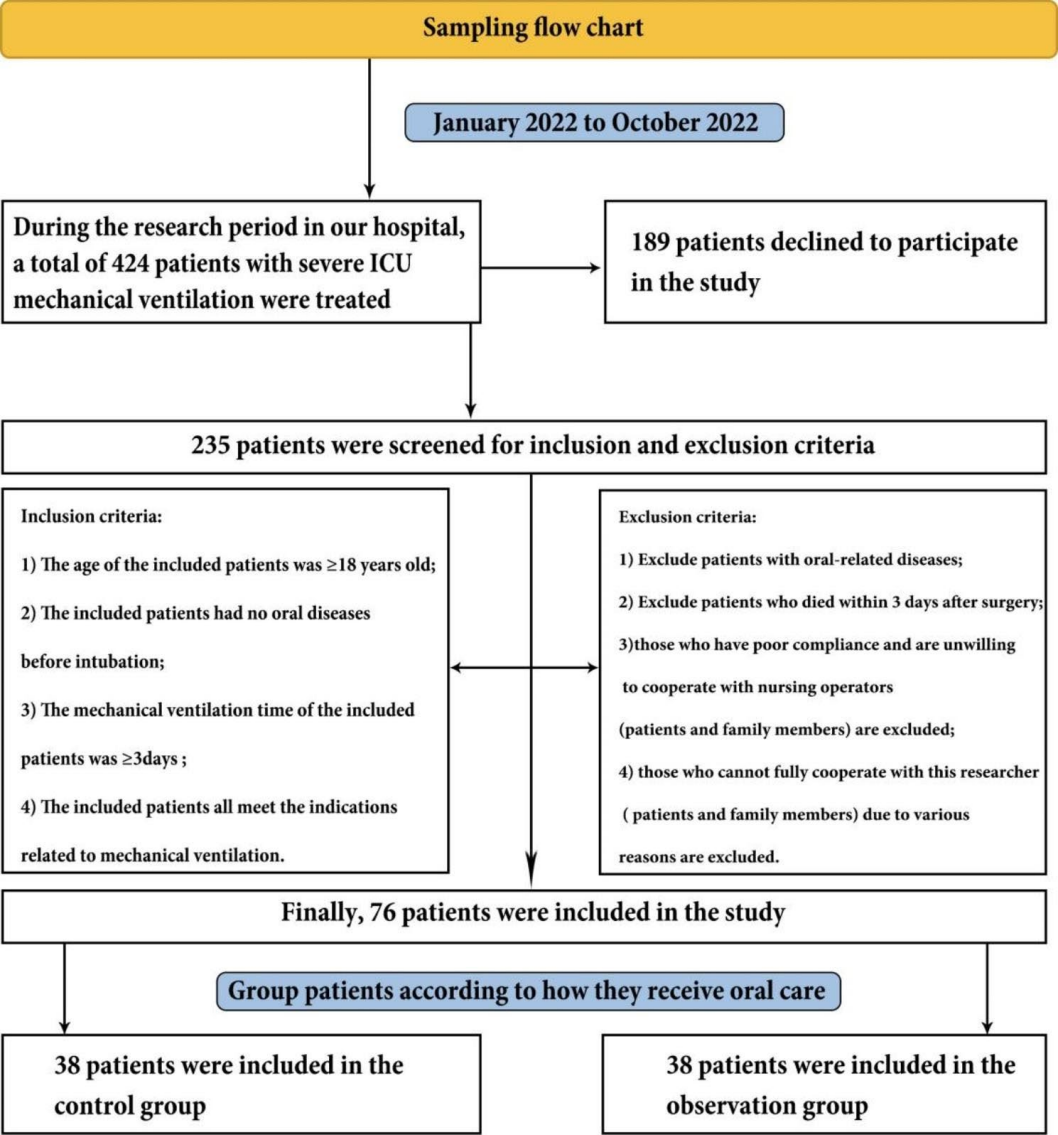
Sampling flow chart

This was a prospective randomized controlled study. A total of 76 critically ill ICU patients with mechanical ventilation who were unconscious and admitted to our hospital between January 2022 and October 2022 were selected as the research objects, and the basic data of the patients were collected using a random table method. Observation group, 38 cases in each group (Fig. 1).

### Inclusion and exclusion criteria

Inclusion: (1) The age of the included patients was ≥ 18 years old; (2) The included patients had no oral diseases before intubation; (3) The mechanical ventilation time of the included patients was ≥ 3days; (4) The included patients all meet the indications related to mechanical ventilation; 4) Patients who are conscious and are willing to cooperate with nursing operators (patients and family members).

Exclusion: (1) Oral-related diseases; (2) Died within 3 days after surgery; (3) those who cannot fully cooperate with this researcher ( patients and family members) due to various reasons are excluded.

The allocation order and secondary outcome data collection dates were generated by the study statistician using a computer-generated randomisation scheme. ICU staff in each group were given 2 months’ notice of the scheduled date of entry into the intervention period.

With a test level of 0.05 for both sides and a test validity of 80%, a sample size estimation method was used for the measurement data of each group. When the sample size for each group was equal, a minimum of 29 cases per group was required. Based on the estimated dropout rate of 15% in the study, a minimum of 36 cases per group was required and a minimum of 72 cases were included in the sample size. The final sample size was 76.

### Method

#### Control group

Patients in the control group received routine nursing intervention: Nursing staff closely monitor the changes in vital signs according to the patient’s condition; at the same time, they also provide patients with psychological, nutritional, diet and other nursing care. Daily oral care during the control period consisted of four daily topical oral chlorhexidine rinses at 0.12% and a modular programme including tooth brushing, oral suctioning and mouth/lip moisturisation tailored to the patient’s needs. At the start of the intervention period, an integrated knowledge translation (iKT) strategy, including point-of-care education, was provided to implement an evidence-based multi-component oral care package. The care package included twice daily (morning and evening) oral assessment and brushing; oral moisturisation, lip moisturisation and removal of secretions every 4 h.

#### Observation group

The patients in the observation group received additional oral comprehensive nursing intervention based on the nursing care provided to the control group. Specific measures included: (1) Secretion cleaning: Regular cleaning of the patient’s mouth to reduce the number of bacteria during the intubation period. Suctioning of oral secretions before turning the patient over to minimize the risk of secretions flowing into the lower respiratory tract. (2) Assisting and promoting expectoration: Helping patients expel sputum through techniques such as turning over and gentle patting on the back. For patients unable to cough up sputum, timely use of appropriate suction techniques to clear respiratory secretions and ensure unobstructed airways. (3) Repeated rinsing and scrubbing: Injecting normal saline into the patient’s mouth while suctioning secretions repeatedly until the aspirated fluid becomes clear. Finally, rinsing the mouth with normal saline and cleaning with a cotton ball. (4) Brushing method: Systematically brushing the patient’s teeth and tongue, paying attention to the inside and outside of the teeth and the occlusal surface. (5) Choice of oral care solution: Using chlorhexidine (0.02%) as the primary oral care solution for patients, and using other solutions like hydrogen peroxide (1.5%), sodium bicarbonate (2.0%), sterilized water (1.0%), and normal saline (0.9%) for those not using chlorhexidine.

### Observation indicators

#### Clinical indicators data

The clinical indicators observed in this research include mechanical ventilation time, ICU stay time, and hospitalization time. These data are recorded by relevant medical staff in our hospital, ensuring accuracy and reliability.

#### Oropharyngeal hygiene status

##### Mouth odor score

The degree of oral odor in patients was evaluated using a visual analogue scale (VAS) on the 1st, 3rd, and 5th days after the implementation of oral care. VAS scores ranged from 0 to 10 points, with higher scores indicating more severe oral odor. Additionally, Oral Chroma® (Nissha FIS, Osaka, Japan) was used to measure the concentrations of three volatile sulfur compounds (hydrogen sulfide (H2S), methyl mercaptan (CH3SH), and dimethyl sulfide) as indicators of oral malodor. VAS is a valid and reliable method for assessing subjective perceptions. The use of Oral Chroma® to measure volatile sulfur compounds provides an objective and quantitative assessment of oral malodor.

##### Plaque index and soft scale index

Dental hygiene status was assessed using the Beck oral score, with scores ranging from 1 to 4 points. Higher scores indicated poorer dental hygiene. The Beck oral score for plaque index and soft scale index is a recognized and validated tool for evaluating dental hygiene status.

##### pH value and blood gas analysis index levels

After the nursing intervention, 5ml of blood samples were collected from each patient to measure pH value and blood gas analysis index levels using a blood gas analyzer. The blood gas analysis indicators included PaO2, SpO2, and PaCO2. Blood gas analysis using a blood gas analyzer is a standard and reliable method for measuring pH value and gas analysis index levels.

##### Occurrence and death of ventilator-associated pneumonia

The occurrence and death of ventilator-associated pneumonia in both groups of patients were recorded by relevant medical staff in our hospital. The diagnostic criteria for ventilator-associated pneumonia included body temperature above 38 °C or below 36.5 °C, positive culture of purulent secretion aspirated from the trachea with the number of colonies ≥ 10^6^ cfu/ml, peripheral blood leukocyte count higher than 10 × 10^9^/L or lower than 4 × 10^9^/L, and chest X-ray showing persistent infiltration. The diagnostic criteria for ventilator-associated pneumonia are widely accepted and commonly used in clinical practice.

### Statistical methods

The graphics software was GraphPad Prism 8; SPSS 25.0 was used to analyze the data; measurement data were compared by t test, $\stackrel{-}{x}$expressed as (± s); categorical data were compared by x² test, expressed as n (%). P < 0.05 means the comparison is statistically significant.

## Results

### Comparison of basic data

Among the 38 patients in the control group, there were 22 males and 16 females; Age between 45 and 87, average age(66. 76 ± 10. 62); Complicated diseases: 10 cases of diabetes, 7 cases of hypertension and 7 cases of hyperlipidemia; Primary disease types: 13 lung diseases, 4 shock, 14 brain diseases, 4 heart diseases, 2 tumors and 1 trauma. Among the 38 patients in the observation group, there were 22 males and 16 females; Age range from 46 to 87, average age 66. 37 ± 10. 24); Complicated diseases: 12 cases of diabetes, 8 cases of hypertension and 9 cases of hyperlipidemia; Primary disease types: 7 cases of lung disease, 4 cases of shock, 18 cases of brain disease, 4 cases of heart disease, 1 case of tumor, 2 cases of trauma and 2 cases of esophageal gastrointestinal bleeding. The baseline data of the two groups were comparable, and there was no significant difference in Table 1 (P > 0 05).

**Table 1:** Comparison of baseline data

	Control group (n = 38 )	Observation group (n = 38 )	t/x²	P
gender				
male	22	22	0.0	0.980
Female	16	16		
age)	45–87	46–87		
Average age (years)	66.76 ± 10.62	66.37 ± 10.24		
Comorbidities				
diabetes	10	12	0.256	0.613
hypertension	7	8	0.083	0.773
Hyperlipidemia	7	9	0.317	0.574
primary disease type				
lung diseases	13	7	2.443	0.188
shock	4	4	0	0.78
Brain diseases	14	18	0.864	0.353
heart diseases	4	4	0	0.670
tumors	2	1	0.347	0.556
Trauma or bleeding	1	2	0.347	0.556

### Comparison of clinical indicators data

The hospitalization time of the two groups was compared (P > 0.05); the mechanical ventilation time and ICU stay time of the observation group were significantly lower than those of the control group (all, P < 0.05). See Table 2 for details.

**Table 2:** Comparison of clinical indicators data

Group (n)	Mechanical ventilation time (h)	Time in ICU (d)	Length of hospital stay (d)
Control group (n = 38 )	159.79 ± 47.64	11.45 ± 6.11	13.13 ± 6.02
Observation group (n = 38 )	101.23 ± 35.27	8.28 ± 5.46	13.34 ± 6.90
t	6.09	2.385	-0.141
P	< 0.001	0.02	0.888

### Comparison of oropharyngeal hygiene

**Fig. 2:**
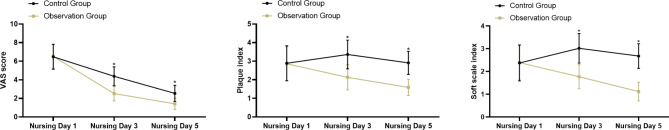
Comparison of oropharyngeal hygiene Note: * indicates P < 0.05 for comparison between groups

As shown in Fig. 2, the oral odor scores of the control group on days 1, 3, and 5 were (6.48 ± 1.34, 4.38 ± 1.02, 2.54 ± 0.87), and the plaque indexes were (2.89 ± 0.94, 3.36 ± 0.77, 2.91 ± 0.62), the soft scale index were (2.38 ± 0.79, 3.02 ± 0.65, 2.68 ± 0.54); the oral odor scores of the observation group on the first, third and fifth day were ( 6.52 ± 1.29, 2.52 ± 0.79, 1.41 ± 0.59), the plaque index was (2.86 ± 0.92, 2.13 ± 0.68, 1.59 ± 0.44), and the soft scale index was (2.39 ± 0.76, 1.77 ± 0.53, 1.12 ± 0.41). The oral odor score, dental plaque index, and soft scale index of the two groups of patients on the first day of nursing intervention were compared (all, P > 0.05); the oral odor score, dental plaque index, and soft scale index of the observation group on the third and fifth day All were significantly lower than those in the control group (all, P < 0.05).

### Comparison of pH value and blood gas analysis indicators

**Fig. 3:**
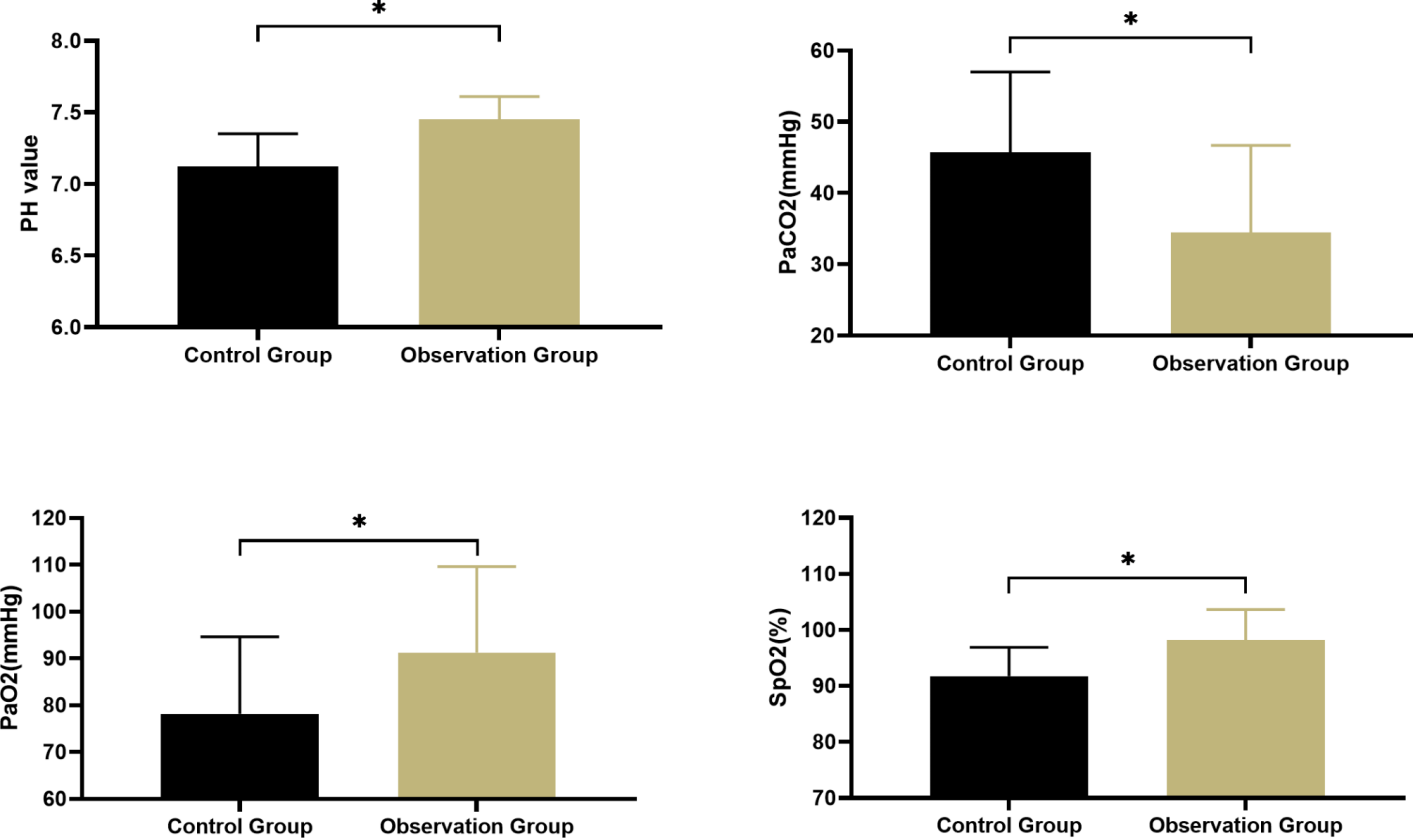
Comparison of various index levels of pH value and blood gas analysis Note: * indicates comparison P < 0.05

As shown in Fig. 3, the pH value, PaCO2 value, PaO2 value and SpO2 value of the control group were (7.12 ± 0.23, 45.73 ± 11.25, 78.19 ± 16.43, 91.78 ± 5.13); the pH value, PaCO2 value, PaO2 value of the observation group The values of SpO2 and SpO2 were (7.45 ± 0.16, 34.49 ± 12.18, 91.27 ± 18.38, 98.23 ± 5.43). The PH value, PaO2 value and SpO2 value of the observation group were significantly lower than those of the control group, while the PaCO2 value was significantly higher than that of the control group (all, P < 0.05).

### Comparison of VAP incidence and mortality

The incidence of VAP in the control group was 55.26%, and the mortality rate was 15.79%. The incidence rate of VAP in the observation group was 21.05%, and the mortality rate was 2.63%. ) See Table 3 for details.

**Table 3:** Comparison of occurrence and death of ventilator-associated pneumonia

Group (n)	Incidence of VAP (%)	mortality rate(%)
Control group (n = 38 )	21 (55.26%)	6 (15.79%)
Observation group (n = 38 )	8 (21.05%)	1 (2.63%)
t	6.09	2.385
P	< 0.001	0.02

## Discussion

Modern medicine believes that the VAP flora is mainly composed of Gram-positive cocci and Gram - negative bacilli, and the proportion of multidrug-resistant bacteria is increasing year by year. Factors related to [9–13]. Mechanical ventilation is the main risk factor for VAP [14]. Studies [15] have shown that the risk of VAP in patients is the highest between 48 h after mechanical ventilation and 48 h after extubation. Another study [16] stated that prevention should be the main focus of VAP diseases, and the main nursing measures are to minimize and reduce the use of ventilators, help patients clear sputum in time, prevent aspiration, food reflux, etc., and prevent bacterial infection. It reproduces in the patient’s mouth ; at the same time, it should also strengthen the patient’s nutritional intake, improve the patient’s immunity, strictly implement aseptic operation, reduce the infection process, cut off the exogenous transmission route, and limit the occurrence of stress ulcers. Reviewing the previous studies [17, 18], the author found that in the clinical routine nursing interventions for ICU patients undergoing mechanical ventilation, most of them only paid attention to basic nursing work, and neglected the hygiene problems of the patients’ oropharynx. Oral comprehensive nursing, as a comprehensive nursing method for oral problems, can effectively make up for the lack of conventional nursing intervention for oropharyngeal problems in ICU patients undergoing mechanical ventilation [19]. The operation of removing microorganisms and dental plaque in the patient’s mouth prevents the risk of complications such as VAP in the patient. Studies [20] have confirmed that oral comprehensive nursing intervention is significantly better than conventional nursing methods in terms of keeping patients’ oral cavity clean, removing plaque and microorganisms.

In this study, 38 ICU mechanically ventilated patients (observation group) were given comprehensive oral care intervention. The results showed that the mechanical ventilation time and ICU stay time of the observation group were significantly lower than those of the control group, and their pH value, PaCO2 value, The improvement effects of PaO2 value and SpO2 value were significantly better than those of the control group, both P < 0.05. The above results were similar to those of previous studies [21]. The body recovers. Some studies [22] pointed out that one of the main risk factors of VAP is the migratory infection of colonized bacteria in the oropharynx and airway of patients. Most patients admitted to the ICU have oral colonization bacteria related to VAP, and these pathogenic bacteria mostly exist in the oropharyngeal secretions of patients, and have the opportunity to cause lung infection along the airway [23]. The results of this study showed that on the 3rd and 5th day of nursing intervention, the oral odor score, dental plaque index, and soft scale index of the observation group were significantly lower than those of the control group, all P < 0.05. Compared with routine nursing intervention, the implementation of oral comprehensive nursing intervention can further improve the oropharyngeal hygiene of ICU patients undergoing mechanical ventilation. At the end of the study, we compared the incidence of VAP and mortality in the two groups. The results showed that the incidence of VAP and mortality in the observation group were significantly lower than those in the control group, both P < 0.05. This result suggests that oral comprehensive nursing intervention can It effectively reduces the incidence of VAP and the risk of death in ICU patients undergoing mechanical ventilation. It is speculated that the reason may be related to the fact that comprehensive oral care intervention can help patients maintain good oral hygiene and reduce the proliferation of bacteria in the patient’s oral cavity.

This trial has several strengths. First, our comprehensive oral care delivery intervention included a low-cost multicentre research collaboration involving discrete ICUs with a broad case-mix, making our results generalisable. Importantly, the total study costs for this trial (excluding investigator costs) were low. By utilising the existing infrastructure, our trial is cost-effective compared to contemporary clinical trials. However, further research on cost-effectiveness is needed to examine the costs of this intervention in practice. Another strength of our study is the use of patient-centred outcomes, and these clinical data are readily available in existing electronic data systems. Our process evaluation provides clinicians and policy makers with clear information about implementation strategies, thereby enhancing interpretation, replication and potentially mitigating uncertainty about negative outcomes.

Several limitations must be considered. First, our final sample size did not reach the number of participants expected in our sample size calculations, and there was insufficient power to detect differences in mortality. Second, we observed differences in patient characteristics between centres over the study period, as would be expected in a stepped wedge-group randomised trial. However, we accounted for this in our analysis by adjusting for centre, time and baseline characteristics, although unmeasured confounders may still be present. Thirdly, due to the implementation of both interventions, we were unable to distinguish between the effects of conventional and implementation of comprehensive oral care on oral health scores. Finally, due to the nature of the interventions, we were unable to blind clinical staff to the study assignment [24–26].

To sum up, the effect of oral comprehensive nursing intervention on patients with ICU mechanical ventilation is significant. The application of nursing intervention can effectively promote the recovery of patients, improve the hygiene problems of patients’ oropharynx, and adjust the levels of pH and blood gas-related indicators in patients. Reduce the risk of VAP occurrence and death in patients.

## Data Availability

The datasets used and analysed during the current study are available from the corresponding author on reasonable request.
